# Editorial: Developments in animal health surveillance, volume II

**DOI:** 10.3389/fvets.2024.1505140

**Published:** 2024-11-26

**Authors:** Bernard J. Phiri, Marta Hernandez-Jover, Arata Hidano, Marta Martinez Aviles

**Affiliations:** ^1^Biosecurity Surveillance, Ministry for Primary Industries, Wellington, New Zealand; ^2^School of Agricultural, Environmental and Veterinary Sciences and Gulbali Institute, Charles Sturt University, Wagga Wagga, NSW, Australia; ^3^London School of Hygiene and Tropical Medicine, University of London, London, United Kingdom; ^4^Centre for Animal Health Research (CISA-INIA/CSIC), Madrid, Spain

**Keywords:** surveillance, biosecurity, animal health, epidemiology, disease, detection, incidence, prevalence

The functions of animal health surveillance include substantiating the absence or distribution of specified disease while facilitating the early detection of exotic or emerging diseases (1). Ideally, surveillance should be timely, sensitive, easy to implement, inexpensive and resource efficient. Fulfilling these functions and attributes is challenging. Specific challenges include limited resources, the ever-lurking threat of disease incursion or spread, underreporting of cases, sustaining motivation for stakeholder participation and the quest to enhance existing surveillance systems. Overcoming these challenges requires continuous effort and novel approaches. Therefore, the goal of the current Research Topic was to collate research-based evidence for solutions or innovative approaches to overcome some of the challenges under different settings.

A useful way to bolster an early warning system is to optimally utilize available resources by using data collected for other purposes. However, the strengths and limitations of such data should be assessed to set the appropriate expectation of their value. Eze et al. examined two utility datasets: a mandatory register (Cattle Tracing System) and a voluntary catalog of fallen stock. Each of the two data sources provided some measure of mortality in the Scottish cattle population. Neither data source was ideal by itself but were complementary. Thus, analyzing and interpreting them in parallel was necessary to produce optimal surveillance outputs. Another possible source of valuable surveillance intelligence is regularly collected on-farm data. In the United States of America, on-farm swine production and disease surveillance data were examined for the prospect of enhancing African swine fever (ASF) surveillance (Schambow et al.). A consultative approach involving a broad range of stakeholders was used to determine the value of the data in enhancing surveillance. Pertinent issues requiring attention to fully realize the value of the data were raised, including data input and sharing, stakeholder expectations, collaboration, labor, and the cost of diagnostic testing. Overall, ordinary on-farm data, along with other types of data, can provide valuable surveillance intelligence when subjected to thorough analysis.

Underreporting of cases is a common phenomenon in many surveillance systems across the world. In Madagascar, underreporting led to the ineffective surveillance of rabies. Resource-deprivation was identified as the main cause of this challenge. Recommended mitigation measures included allocating more resources and/or better utilization of existing ones. For instance, a One-Health approach could be used to pool resources from the veterinary and public health sectors (Dreyfus et al.; Andriamandimby et al.). In France, an innovative holistic and inter-sectoral framework optimized the usage of available resources to enhance the efficiency of animal health surveillance. This was achieved by forming platforms composed of experts from different industries (Dupuy et al.). Newer technology can also help in using available resources efficiently. The Haiti national rabies surveillance program adopted an electronic application for managing its integrated bite case management in 2018. Previously, bite case management was paper based. The newer technology led to superior data quality, improved data completeness, and shortened durations for notifications. Overall, the timeliness of surveillance improved because the flow of data and analysis were quicker compared to the paper-based system. These gains were achieved with minimal increase in operational costs (Schrodt et al.).

Movement of animals is a common pathway for disease spread. Hence, in-depth knowledge of animal movement patterns is very informative in developing targeted surveillance and disease control measures. A description of trade networks for cattle, small ruminants and pigs in Uganda provided useful insights for this purpose. The networks were derived from the 2019–2021 data for animal movement permits. The findings highlighted key nodes that could be targeted to enhance surveillance and inform decision-making regarding infectious animal disease control (Hasahya et al.). Similarly, an analysis of factors influencing seasonal peaks and regional movement patterns of beef cattle in Japan provided useful biosecurity insights. The findings could inform the development of risk-based surveillance measures suited for specific age groups, regions, and seasons (Murato et al.). Identifying influential nodes or factors along disease transmission pathways allows for the application of resources where they will maximize surveillance sensitivity.

Keeping stakeholders actively engaged and participating in surveillance to maintain a vibrant early warning system can be challenging. The Canadian dairy network for antimicrobial stewardship and resistance tackled this difficulty by allowing farmers and veterinarians to visualize data online. Metrics for antimicrobial use were benchmarked in relation to antimicrobial resistance and animal health in dairy herds. This allowed comparisons among participant farms with the view of enhancing antimicrobial stewardship practices on dairy farms in Canada (Fonseca et al.). However, similar bidirectional communication with stakeholders needs to be well-managed. For instance, in situations where outbreak alerts are communicated to stakeholders, the quality and frequency should be well-balanced. Frequent alerts that are not meaningful or irrelevant to stakeholders can damage the credibility of the surveillance system and diminish stakeholder participation. In the United Kingdom, veterinary practitioners were consulted in selecting notification thresholds that were clinically relevant for detecting genuine outbreaks of canine disease (Tamayo Cuartero et al.). Unimpeded communication between surveillance operators and those involved more directly with animals is invaluable for both detecting outbreaks early and influencing appropriate biosecurity practices.

Timely production of surveillance outputs is vital for the success of disease control efforts, particularly for fast-spreading diseases like foot and mouth disease (FMD). Ellis et al. provide evidence that intensive environmental sampling could detect FMDV in a herd more quickly than clinical inspection. Adopting this technique could drastically reduce the cost of FMD control. The authors also evaluated, using a mathematical model, if at-risk farms could be monitored using environmental sampling instead of resorting to pre-emptive culling so that the number of animals culled may be reduced to minimize the socio-economic impact on farmers. Similarly, machine-learning based technology can enhance early detection. The performance of video surveillance system in detecting lameness in dairy cattle was found to be comparable to that of two experienced veterinarians in the United Kingdom. Additionally, the video system was more sensitive than a trained veterinarian in detecting painful foot lesions (Anagnostopoulos et al.). Use of smart technology could not only improve the efficiency of surveillance but also free up resources like veterinary practitioners to perform other functions.

The articles in the current Research Topic provide valuable contributions to the pool of alternatives for improving animal health surveillance. These alternatives include using available resources more holistically, such as adopting a One-Health or inter-sectoral approach. Underreporting can be minimized by implementing surveillance methods that are easy to apply, and the use of smart technology is a feasible option for this purpose, provided it is accepted by key stakeholders. Gaining insights into disease transmission pathways, along with their temporal and spatial influencing factors, enables the implementation of risk-based surveillance. It also allows for the deployment of resources where they are most needed, maximizing the benefits. Maintaining social license and fostering productive stakeholder engagement are crucial for an early warning system to function effectively. Demonstrating to stakeholders that their contribution is valued and relevant is essential in this regard. Practical steps include allowing visualization of a broad perspective derived from the aggregated data that is relevant or helpful to stakeholders. For instance, visualizing geographical variations across a country or having the ability to benchmark farm performance against the geographical averages for variables such as disease incidence or drug usage. Ultimately, animal health surveillance systems around the world have similar functions but vary in capacities and capabilities, yet all aspire to improve.
